# The Viable but Non-Culturable (VBNC) State, a Poorly Explored Aspect of Beneficial Bacteria

**DOI:** 10.3390/microorganisms12010039

**Published:** 2023-12-25

**Authors:** Laura Abisaí Pazos-Rojas, Alma Cuellar-Sánchez, Ana Laura Romero-Cerón, América Rivera-Urbalejo, Pieter Van Dillewijn, Diego Armando Luna-Vital, Jesús Muñoz-Rojas, Yolanda Elizabeth Morales-García, María del Rocío Bustillos-Cristales

**Affiliations:** 1 Faculty of Stomatology, Meritorious Autonomous University of Puebla (BUAP), Puebla 72570, Mexico; laura.pazos@correo.buap.mx (L.A.P.-R.); america.rivera@correo.buap.mx (A.R.-U.); 2 Monterrey Institute of Technology, School of Engineering and Sciences, Monterrey 64700, Mexico; alma.cuellar@tec.mx (A.C.-S.); a01736238@tec.mx (A.L.R.-C.); dieluna@tec.mx (D.A.L.-V.); 3 Department of Environmental Protection, Estación Experimental del Zaidín, Consejo Superior de Investigaciones Científicas, 18008 Granada, Spain; pieter.vandillewijn@eez.csic.es; 4 Ecology and Survival of Microorganisms Group, Laboratory of Microbial Molecular Ecology (LEMM), Center for Research in Microbiological Sciences, Institute of Sciences, Meritorious Autonomous University of Puebla (BUAP), Puebla 72570, Mexico; jesus.munoz@correo.buap.mx; 5 Faculty of Biological Sciences, Meritorious Autonomous University of Puebla (BUAP), Puebla 72570, Mexico

**Keywords:** VBNC state, stress, beneficial bacteria, rhizosphere, latency, survival

## Abstract

Many bacteria have the ability to survive in challenging environments; however, they cannot all grow on standard culture media, a phenomenon known as the viable but non-culturable (VBNC) state. Bacteria commonly enter the VBNC state under nutrient-poor environments or under stressful conditions. This review explores the concept of the VBNC state, providing insights into the beneficial bacteria known to employ this strategy. The investigation covers different chemical and physical factors that can induce the latency state, cell features, and gene expression observed in cells in the VBNC state. The review also covers the significance and applications of beneficial bacteria, methods of evaluating bacterial viability, the ability of bacteria to persist in environments associated with higher organisms, and the factors that facilitate the return to the culturable state. Knowledge about beneficial bacteria capable of entering the VBNC state remains limited; however, beneficial bacteria in this state could face adverse environmental conditions and return to a culturable state when the conditions become suitable and continue to exert their beneficial effects. Likewise, this unique feature positions them as potential candidates for healthcare applications, such as the use of probiotic bacteria to enhance human health, applications in industrial microbiology for the production of prebiotics and functional foods, and in the beer and wine industry. Moreover, their use in formulations to increase crop yields and for bacterial bioremediation offers an alternative pathway to harness their beneficial attributes.

## 1. Introduction

Bacteria in the VBNC state exhibit a remarkable phenomenon: they are unable to grow and form colonies on conventional culture media, yet they remain alive and able to restart their metabolic activity [1]. Cells in this status typically display reduced levels of metabolic activity and undergo significant metabolic alterations, such as reductions in nutrient transport and respiration rates and macromolecular synthesis, and form resistance structures similar to spores [2,3,4,5]. However, a feature that distinguishes the VNBC state is the continuous gene expression within these cells [3].

**Figure 1:**
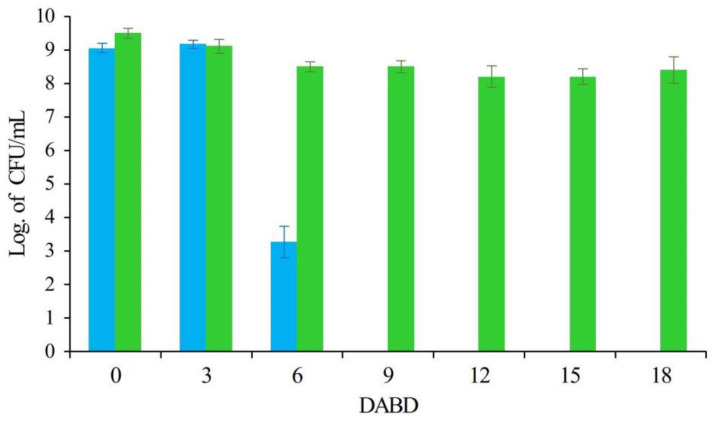
CFU/mL (log) of *Pseudomonas putida* KT2440 under desiccation stress at 18 days after the beginning of desiccation (DABD), in the presence (green bars) and absence (blue bars) of trehalose. Adapted from Pazos-Rojas et al., 2019 [4].

A typical response of cells entering into the VBNC state is shown in Figure 1, under stressful conditions caused by desiccation in the presence and absence of a cytoprotective agent such as trehalose. In presence of trehalose, bacterial cells strongly avoid entry into the VBNC state. Conversely, in the absence of the protector, a decrease in the number of CFUs/mL is observed under the same environmental stress. At 9 days after the beginning of desiccation (DABD), the cells enter into the VBNC state and remain so until 18 DABD, which has been confirmed using several methodologies [4]. Interestingly, after prolonged rehydration or rapid rehydration in the presence of plant exudates, these bacteria reach high numbers, indicating that they have returned to the culturable state [4].

### Conditions That Induce VBNC State

Bacterial cells commonly respond to stressful conditions by losing their ability to form colonies in standard culture media, although cells can remain viable for long periods of time [5]. Cells may enter into the VBNC state in response to natural stresses, such as starvation, extreme temperatures, high osmotic or oxygen concentrations, or exposure to white light [1]. In general, extreme environmental conditions can be lethal unless they adopt a latency status; for example, it is known that one of the most critical factors for bacterial survival is the availability of nutrients in the surrounding environment. When bacterial cells are subject to nutrient starvation conditions, they may reduce their size and become more resistant to adverse environmental conditions; alternatively, they could induce a latent estate, forming viable but non-culturable cells or structures resembling spores [3]. However, spore-forming bacteria are typically not classified within the VBNC state literature [3]. It is interesting that not only environmental stress can induce the VBNC state, but various processes and bactericidal substances are able to induce this state as well. For example, milk pasteurization [6], wastewater chlorination [2], and the use of food preservatives such as potassium sorbate and sodium benzoate [7] have been documented as inducers of the VBNC state.

## 2. Bacterial Species Entering the VBNC State

A substantial portion of the bacterial species known to enter the VBNC state include human pathogens such as *Campylobacter* spp., *Escherichia coli* (EHEC strains), *Francisella tularensis*, *Helicobacter pylori*, *Legionella pneumophila*, *Listeria monocytogenes*, *Mycobacterium tuberculosis*, *Pseudomonas aeruginosa*, several species of *Salmonella* spp., *Shigella* spp., and numerous pathogens from the genus *Vibrio* sp., with *Vibrio vulnificus* being one of the most studied in terms of the VBNC state [2].

The list of pathogenic bacteria that can adopt the VBNC state as a survival strategy includes pathogens affecting not only humans but also animals, such as *Photobacterium damselae*, infecting fish [8]; *V. vulnificus*, an eel pathogen [9], and *V. shiloi*, which causes the bleaching of corals [10,11]. Several plant pathogens have been described—for example, *Ralstonia solanacearum* in tomato plants [12]; *Xanthomonas axonopodis* colonizing grapefruit plants [13]; *Erwinia amylovora* infecting ripe apples [14]; *Pseudomonas syringae* in tomato, cereals, almond, cherry, and plum plants; *Acidovorax citrulli* infecting a wide variety of Cucurbitaceae, causing bacterial spot disease (bacterial fruit blotch) [15]; and *Agrobacterium tumefaciens*, which causes tumors in different dicotyledons.

Since the publication of Xu’s et al. study [5] more than 30 years ago, a substantial body of research around the world has focused on documenting the occurrence of the VBNC state in different bacterial species [2,4,16,17,18,19,20,21,22,23,24,25,26].

To date, approximately 101 bacterial species spanning 50 different genera have been reported to exhibit the VBNC phenomenon [2,18,25,26,27]. In the present review, we analyze the beneficial bacterial species where the VBNC status has been reported and the importance of this within their potential in areas such as biotechnology, agriculture, and the food industry. For this review, a comprehensive search of publications was conducted utilizing Web of Science, PubMed, and Google Scholar. The research by Oliver (2010) [27] and Dong (2019) [25], who previously compiled a list of bacteria entering the VBNC state under specific conditions, served as a reference. The search terms included “viable but non-culturable bacteria” or “viable but non-culturable state” or “beneficial bacteria in VBNC state”. A specialized search was conducted for each bacterial species included in the review. This involved using the scientific name of each species along with specific terms such as “biotechnological applications”, “beneficial applications”, “VBNC”, or “viable but non-culturable state in…”. The bibliographic search encompassed the period between 1982, the year in which the concept of the viable but non-culturable state was introduced by Rita Colwell [2,5], and 2023.

## 3. The VBNC State in Beneficial Bacteria

Beneficial bacteria play a crucial role in maintaining life on our planet. Some realize nitrogen fixation [28], mineral solubilization [29], and greenhouse gas consumption and are thus viewed as gatekeepers preventing excessive methane emissions from escaping the atmosphere [30]. Due to the several processes in which beneficial bacteria participate, they have been used to increase crop production [31]. Some beneficial properties of these bacteria include plant growth promotion [32,33], the control or inhibition of the activity of plant pathogens [34,35], improvements in soil structure, bioaccumulation, or the microbial leaching of inorganics [36], the bioremediation of xenobiotic compounds [37,38], or the production of compounds of industrial interest [39,40].

Certain bacteria that interact with their hosts establish mutually beneficial relationships. Probiotics, for instance, interact with humans to improve overall health. They can eliminate or remove pathogens [41], reinforce the epithelial barrier, and induce the migration of fibroblasts and epithelial cells [42]. In the immune system, probiotics are related to the modulation and activation of intraepithelial lymphocytes, natural killer cells, and macrophages through the induced production of cytokines [43].

On the other hand, there are beneficial bacteria with industrial applications that favor the production of certain foods, prebiotics, and beverages. In the brewing and wine industry, bacteria play a crucial role in the fermentation process. Lactic acid bacteria (LAB) and yeasts are instrumental in this context. LAB catalyze the conversion of dicarboxylic malic acid into monocarboxylic lactic acid and carbon dioxide (malolactic fermentation MLF) and yeasts convert sugars into alcohol (alcoholic fermentation) [44]. During malolactic fermentation by LAB, no free intermediary products are formed, achieving a more palatable wine by reducing the tart taste of malic acid. Additionally, malolactic fermentation reduces the amount of residual nutrients available to support microbial growth, enhances the wine aroma, improves the microbial stability, and reduces the acidity of wine, making the wine more stable before being bottled [45]. Despite the crucial importance and diverse benefits of bacteria across different levels, our comprehension of beneficial bacteria in the VBNC state remains significantly limited. The progress in this field has primarily concentrated on pathogenic bacteria due to their profound impact on human health.

Table 1 shows the beneficial bacteria reported so far to enter the viable but non-culturable state under different conditions. This table displays a variety of conditions that can induce the VBNC state and the respective taxonomic groups to which each described species belongs.

**Figure 2:**
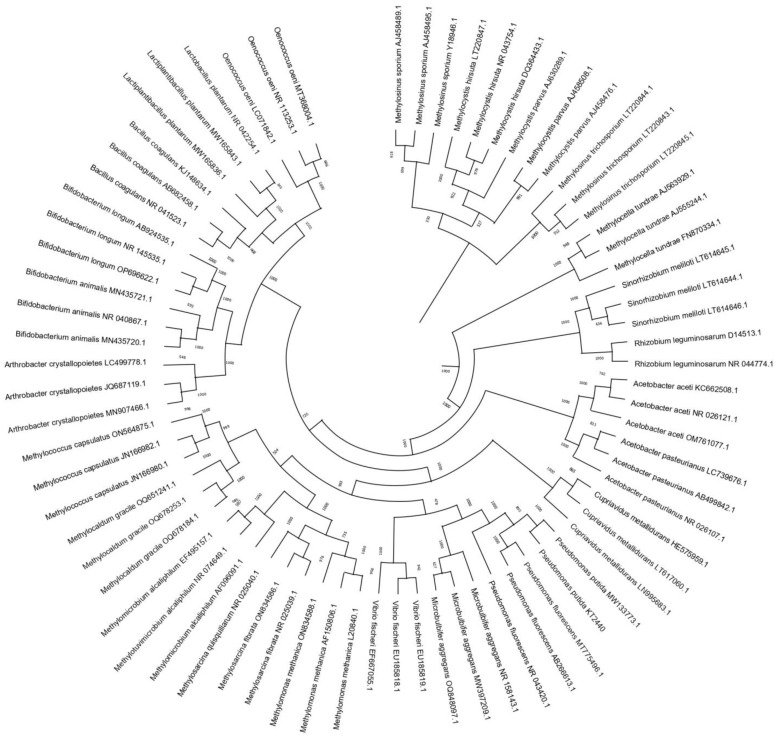
Phylogenetic tree of beneficial bacterial species entering the VBNC state. Phylogenetic trees were constructed by the neighbor-joining method [58] using Clustal X 2.1, BioEdit 7.7, and Mega 4 ©1993–2011 software. A bootstrap confidence analysis was applied on 1000 replicates to determine the reliability of the topology obtained.

The phylogenetic relationships between different beneficial bacterial species reported to enter the VBNC state were analyzed (Figure 2). The sequences were compared with the data available in the National Center for Biotechnology Information (NCBI) database. To evaluate viable but unculturable strains, phylogenetic trees were constructed by the neighbor-joining method [58] using the Clustal X 2.1, BioEdit 7.7, and Mega 4 ©1993–2011 software. A bootstrap confidence analysis was applied on 1000 replicates to determine the reliability of the topology obtained [59]. The phylogenetic tree showed that these bacteria are very diverse. The furthest phylogenetic group corresponds to *Methylosinus*, *Methylocystis*, and *Methylocella*. The genera *Rhizobium*, *Sinorhizobium*, and *Acetobacter* are groups that are phylogenetically closer to each other. *Methylococcus*, *Methylocaldum*, *Methylomicrobium*, *Methylotuvimicrobium*, *Methylosarcina*, and *Methylomonas* are phylogenetically close to *Vibrio*, *Microbulbifer*, and *Pseudomonas*. Another group that can be identified is formed by *Arthrobacter*, *Bifidobacterium*, *Bacillus*, *Lactobacillus*, and *Oenococcus*, with some strains being phylogenetically closely related and others being very distant species. When analyzing the phylogenetic relationships, it can be observed that the viable but non-culturable state is not exclusive to any taxonomic group or group of species, and it is a widely distributed strategy in phylogenetically close and distant species.

The beneficial species in which the viable but non-culturable state has been reported are described below, highlighting their main applications in different areas, such as biotechnology, agro-biotechnology, health, and industrial applications.

### 3.1. Alphaproteobacteria and the VBNC State

Within the group of Alphaproteobacteria that enter the VBNC state are organisms belonging to the genera *Acetobacter*, *Methylosinus*, *Methylocistis*, *Methylocella*, *Rhizobium*, and *Sinorhizobium* (Table 1).

*Acetobacter aceti* and *Acetobacter pasteurianus* have a great relevance in vinegar production, since they can transform ethanol into acetic acid through oxidative fermentation [60]. The VBNC state in *A. aceti* has been documented in wine production [46], and *A. pasteurianus* enters the VBNC state under high acid stress generated during the fermentation process [47]. Studies are still lacking that support the idea that *A. aceti* and *A. pasteurianus*, under a non-culturable state, can follow the fermentation process, which would help to increase production. The ability of these species to persist under adverse conditions represents a challenge in developing novel ways to improve industrial yields, enhance wine quality, and drive vinegar production on a larger scale.

The methylotrophic bacteria *Methylosinus sporium*, *Methylosinus trichosporium*, *Methylocystis hirsuta*, *Methylocystis parvus*, and *Methylocella tundrae* are methanotrophic microorganisms, also called methane-oxidizing bacteria (MOB), capable of generating energy through the oxidation of methane gas [61]. MOB have different biotechnological applications, mainly the biological mitigation of the methane greenhouse gas, the production of high-value products from methane, and the bioremediation of pollutants [62,63]. Because of the ecological importance of these microorganisms, it is important to understand how different conditions can affect methane consumption. One of the conditions that induces the VBNC state in MOB is freeze-drying and cryopreservation (Table 1). This represents a challenge to researchers, because culturable cells are needed for beneficial application. Most studies focus on how to avoid the loss of cultivability, but it is likely that MOB can return to a culturable state when in an environment with methane gas. This has been observed with different pathogen species of bacteria that, when found in favorable conditions, leave the non-culturable state [27]. Research with this group of bacteria in the VBNC state is scarce, representing a challenge in understanding this survival strategy. In the future, this could stimulate these organisms to consume methane, aiding in the degradation of pollutants and the production of high-value biomass.

Other species of Alphaproteobacteria are *Sinorhizobium meliloti* and *Rhizobium leguminosarum*, found mainly in the soil, which have acquired, by horizontal gene transfer, the ability to associate in symbiosis with leguminous plant roots. The association of rhizobia and legumes occurs through a complex signaling process that generates nodules, organs specialized in the fixation of atmospheric nitrogen [64]. Biological nitrogen fixation is a vital process in agriculture, allowing the production of nitrogen through legume–rhizobium symbiosis, which contributes to increased nitrogen levels in the soil, resulting in increased plant growth [65]. In addition to the ability to fix nitrogen, *R. leguminosarum* isolated from fava bean root nodules was shown to generate an induced systemic response against infection by bean yellow mosaic virus [66]. The VBNC state in this bacterium is related to the presence of cupric sulfate (Table 1). For *S. meliloti*, the factors that induce the VBNC state are temperatures of 20 °C to 25 °C, incubation under anoxic conditions, and incubation in nitrocellulose filters at low relative humidity (Table 1). It is interesting that, under these conditions, the bacteria can persist. This could explain their survival capacity under this type of stress in the environment. The importance of rhizobia for agriculture highlights the need to learn more about their beneficial functions in the VBNC state, because it is unknown whether, under a non-culturable state, bacteria can continue performing nitrogen fixation, generate an induced systemic response, or even develop nodules.

### 3.2. Betaproteobacteria and the VBNC State

In the group of Betaproteobacteria, the only beneficial bacterium where the VBNC state has been documented is *Cupriavidus metallidurans*. This bacterium is metallophilic, found in environments containing high concentrations of heavy metals and industrial wastes rich in toxic heavy metals, often mixed with recalcitrant organic compounds and hydrocarbons [67]. It is an ideal bacterium for bioaugmentation purposes in environmental applications due to its strong resistance to environmental stress factors and its adaptation capacity [68]. Another specific application is its ability to convert gold chloride into 24-carat gold in one week [69]. These observations suggest that bacteria actively contribute to the formation of gold grains in surface environments [70]. With this bacterium, the conditions that can induce its loss of cultivability have been studied, as well as the conditions that can return it to a cultivable state. The addition of water and gluconate is sufficient for *C. metallidurans* to be culturable in 24 h [49]. Due to its interesting applications and ability to persist in toxic environments, it is an excellent model to study the mechanisms of coping with heavy metal stress. This unique ability to metabolize toxic substances and enter the VBNC state without water and carbon sources could help us to understand how the origin of life occurred.

### 3.3. Gammaproteobacteria and the VBNC State

One of the groups with the most beneficial species in which the VBNC status has been demonstrated is Gammaproteobacteria. *Methylomonas methanica*, *Methylosarcina fibrate*, *Methylocaldum gracile*, *Methylomicrobium alcaliphilum*, and *Methylococcus capsulatus* are methylotrophs. This type of bacteria uses methanol and methane as the only carbon source [71]. Methylotrophs can inhabit soil, water, and plants, powering the carbon cycle [72]. When *M. fibrate* was co-inoculated with species of *Methylomonas* and *Cupriavidus taiwanensis* LMG 19424, its growth was highly stimulated [73]. Moreover, methane oxidation was elevated when methanotrophs interacted with algae and moss [74]. Cryopreservation is one method that induces the VBNC state in methanotrophs (Table 1). The study of the VBNC state in methanotrophs is essential due to its role in the carbon cycle and therefore in the planet’s life. It is relevant to consider that interaction with other microorganisms or with plants could be an alternative strategy for these microorganisms to return to the cultivable state, being able to continue exerting their beneficial effects.

*Microbulbifer aggregans*, another Gammaproteobacteria species, is a halophilic, Gram-negative bacterium isolated from sediment in the Matang mangrove forest, Malaysia [75]; its importance is centered on its capability to reduce sulfur due to the presence of genes involved in this process [50]. Sulfur is an essential element for life and is present in amino acids, proteins, enzymes, vitamins, and other biological molecules [76]. *M. aggregans* is a rarely explored bacterium. Recently, it was observed that non-culturable cells exhibited a change in cell shape from rod to coccus, and the genes responsible for sulfate reduction were upregulated in the VBNC state [50]. These findings demonstrate this species’ importance in the environment in which it lives, because the effective reduction of sulfur in the non-culturable state may occur in response to changes in the sulfur concentration in the environment, playing a relevant role in the sulfur cycle in marine environments.

Within the Gammaproteobacteria, there is also the genus *Pseudomonas*. The species of this genus have a great capacity to use different nutrients as carbon sources, which explains their ubiquity. Their enzymatic activity makes them an important group of microorganisms responsible for the aerobic degradation of many compounds in different ecosystems [77]. Some species of *Pseudomonas* can promote plant growth by suppressing pathogenic microorganisms, synthesizing growth-stimulating plant hormones, and promoting increased plant disease resistance [78]. *Pseudomonas fluorescens* and *Pseudomonas putida* KT2440 are bacteria able to colonize plants’ roots and promote their growth. Some strains of *P. fluorescens* have been shown to degrade a variety of organic compounds, thus being important in bioremediation [79]. This bacterium is used for biocontrol to protect plants against soilborne fungal pathogens. One mechanism for the biocontrol by *P. fluorescens* is the ability to produce antibiotics [80]. It also stimulates induced systemic resistance (ISR) [81] and the production of volatile compounds [82].

On the other hand, *P. putida* KT2440 is a bacterium capable of using different aromatic compounds as a carbon source [83]. Metabolizing xenobiotic compounds, it can colonize the roots of plants such as corn, wheat, strawberry, sugarcane, and spinach [84] and is capable of promoting the growth and health of plants [85,86]. *P. putida* KT2440 has been used in various bacterial formulations to enhance plant growth [86,87].

The potential of *P. putida* KT2440 and *P. fluorescens* to exert beneficial effects can be affected by their exposure to different stresses that induce the VBNC state, such as saline environments for *P. fluorescens* and desiccation for *P. putida* KT2440 (Table 1). However, it has been shown that plant–bacteria interaction is a mechanism that allows these bacteria return to the cultivable state. Therefore, their use in the formulation of stable bacterial inoculants that can stimulate plant growth after rehydration [4], or the development of formulations to perform biological control [23], could be an alternative to reduce the overuse of nitrogen fertilizers and pesticides, decreasing the damage caused by these chemical products [88].

*Vibrio fischeri*, also belonging to the Gammaproteobacteria, is a luminous marine bacterium that lives freely or in symbiosis with different species of fish and squid [89]. The most studied interaction in this microorganism is the symbiosis with the Hawaiian squid, *Euprymna scolopes*, inducing bioluminescence that the squid uses to avoid predation during nocturnal activity [90]. In the squid–*Vibrio* symbiosis, bacteria are found in a ventral tissue called the lumen organ. The relationship between *V. fischeri* and *E. scolopes* is characterized by daily rhythmic cycles that control the population dynamics of bacteria. At night, when squid take feed, the light organ fills with bioluminescent *V. fischeri*. At dawn, the squid bury themselves in the sand and ventilate approximately 90% of the bacterial population to the environment; the remaining bacteria repopulate the crypts and are ready to produce light at dusk [91]. This example of symbiosis shows beneficial microorganisms’ important role in their hosts’ health and activities. The role of the VBNC state in *V. fischeri* living freely in the marine environment or in symbiosis is still unknown. It has only been discovered that non-culturable cells lose their luminescence in response to fluctuations in salinity [52]. The loss of luminescence could have relevance in the diagnosis of marine environments, allowing the determination of alterations in salinity, temperature, and the concentrations of nutrients related to the number of luminescent bacteria of *V. fischeri*, with the interpretation that the fewer luminescent bacteria observed, the greater the disturbance present in the marine environment.

### 3.4. Actinobacteria and the VBNC State

The Actinobacteria group includes *Sinomonas albida*, formerly known as *Arthrobacter albidus*, isolated from a seep substrate composed of volcanic rock from Niigata, Japan [92]. Among the main applications of this microorganism, it has a demonstrated ability to exit the VBNC state in the presence of the RpF protein, along with its efficient flocculant activity [55]. This flocculent activity represents a possible application in wastewater treatment and residual sludge dewatering. At present, little is known about this species, which represents an alternative and challenge in terms of taking advantage of the biotechnological properties that *S. albida* can provide.

The presence of bacteria in the human digestive tract offers various properties related to health, such as the regulation of intestinal microbial homeostasis, the inhibition of pathogenic bacteria, the modulation of the immune response, anticancer effects, the production of bacteriocins, or the bioconversion of diet components into bioactive compounds [93]. Recently, the production and consumption of products with beneficial strains for human health has increased considerably [94]. *Bifidobacterium longum* and *Bifidobacterium animalis* subsp. *lactis* are multifunctional probiotic Actinobacteria with clinical effectiveness, including immunomodulatory, anti-inflammatory, antimutagenic, and anticancer properties and alleviating gastrointestinal diseases [94]. In both species, it has been observed that their storage at low temperatures in fermented foods can cause a loss of cultivability (Table 1). In this respect, the determination of the viability and activity of probiotic bacteria is of great economic, regulatory, and technological importance to ensure that fermented products or formulations with probiotics carry bacteria capable of exerting their beneficial effects.

### 3.5. Firmicutes and the VBNC State

*Bacillus coagulans* is a species of Firmicutes that is considered probiotic-safe. This bacterium has the ability to endure high temperatures and it has developed genetic stability through several years of commercial production [95]. Its main benefits for human health include the modulation of gastrointestinal disorders, immune system stimulation, and lowering cholesterol [96]. This bacterium can form endospores and survive for decades in unfavorable environmental conditions. It was previously thought that its high persistence was due only to its ability to form spores; however, it has been observed that *B. coagulans* can enter the VBNC state as a strategy to face adverse conditions [57]. Little is known about the return to cultivability when humans consume these beneficial microorganisms. Some studies have shown that bacteria secrete certain specific proteins to exit the latency state [97], which is a possibility in the case of *B. coagulans*. This represents a task for future research. If we consider that some beneficial bacteria are capable of returning to the culturable state when they interact with their hosts [4], it is very likely that, although culturable bacteria are not detected in fermented foods, when they are consumed by humans, the conditions present in these hosts may favor their return to a cultivable state, being able to exercise their beneficial properties.

*Lactiplantibacillus plantarum* is a Firmicutes species widely distributed in various environments, such as the gastrointestinal, vaginal, and urogenital tracts, and in dairy products, vegetables, meat, hay, and wine. This ability to adapt to different conditions demonstrates its metabolic diversity [98], having potential for various applications. The main applications of *L. plantarum* include the fermentation of foods such as cheese, kefir, sauerkraut, fermented meat products, fermented vegetables, and beverages [99]. It has been reported that *L. plantarum* produces antimicrobial substances such as plantaricin [100] and can remove microcystins, the main toxins produced by cyanobacteria [101], which makes it an alternative food preservative or a compound to fight infections. Studies of the VBNC state in *L. plantarum* have reported that it can remain latent during beer storage, which contributes to the deterioration of this beverage [20]. Another study showed that *L. plantarum* in the VBNC state can inhibit microcystins, which contributes to the preservation of fermented foods [101]. It is interesting to observe that the VBNC state in this species can be detrimental for beer production but beneficial for food preservation, reflecting the versatility of non-culturable *L. plantarum* to continue performing its functions. This suggests that when used as a probiotic to improve human health, it can provide its benefits without being culturable. In short, it is a microorganism that should be studied in greater depth, particularly regarding what happens during the VBNC state, to take advantage of its diversity of applications.

In the production of alcoholic beverages, the involvement of microorganisms is crucial for successful fermentation. A significant challenge in the brewing and winemaking industry is the spoilage of wine and beer by lactic acid bacteria, which have the ability to enter the VBNC state [20]. *Oenococcus oeni* is a Firmicutes bacterium, belonging to the lactic acid bacteria group, adapted to the stressful environment of wine. It is widely used as a starter microorganism to carry out malolactic fermentation (MLF), where L-malate is converted into L-lactate [102]. The use of starter cultures of *O. oeni* remains difficult in some wine regions, due to the hostile environment created by the low pH and the presence of SO_2_ and ethanol [103]. The ability of *O. oeni* to respond these stress conditions has great relevance in terms of increasing wine production at a lower cost. Thus far, knowledge of the VBNC status of *O. oeni* is limited. It is known that the presence of sulfur dioxide induces the loss of cultivability, and the addition of arginine to the medium allows a return to the cultivable state [102]. According to findings with *O. oeni*, although the bacteria are in the VBNC state, they may still be capable of carrying out malolactic fermentation, although perhaps not as efficiently as their cultivable counterparts. When cells are present in a rich environment and arginine is added, the bacteria return to the cultivable state, increasing the fermentation efficiency. The generation of new knowledge requires changing the paradigm and legislation that require all microorganisms used in food production to be culturable. It is feasible that the use of non-culturable cells could decrease production costs because expensive infrastructure would not be required to preserve the bacteria, since they have all the necessary equipment to preserve themselves.

The VBNC state could be an important reservoir of beneficial bacterial species, as this state constitutes a survival strategy in response to harsh environmental conditions. The capability of bacteria to enter the VBNC state in response to stress could have important biotechnological applications. It is possible consider that the VBNC state may reduce negative selection and regulate microbial dominance in soil, as the rhizosphere, where plants could induce a selective bacterial revival by releasing selected organic compounds, directly influences the diversity present in these environments. In the same way, the hosts themselves could be exerting a selection bias by releasing compounds that determine the viability of certain bacteria necessary in a given physiological state.

## 4. Techniques to Evaluate Bacterial Viability in the VBNC State

**Figure 3:**
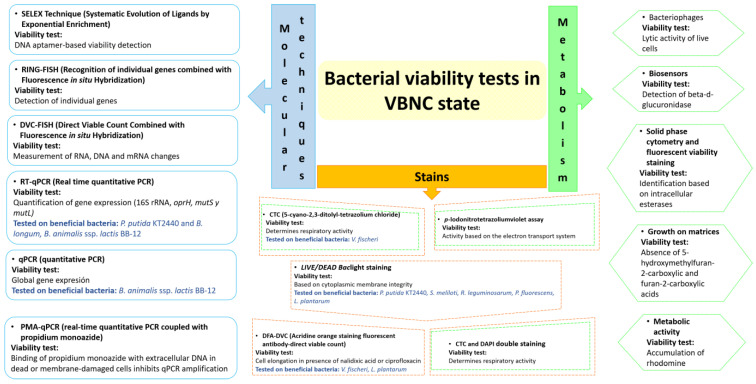
Methodologies for determination of bacterial viability in the VBNC state. Molecular biology-based techniques are highlighted in blue. Methods reliant on identifying metabolic activity are represented in green. Techniques employing dyes for cell staining are depicted in orange. The orange and green methodologies involve techniques for staining and the identification of metabolic activity. The blue text shows beneficial microorganisms and the techniques that have been used to study their VBNC state.

Currently, several methodologies have been reported that can be used to determine the viability of a bacterium under the VBNC state (Figure 3). The methodologies are divided into three groups: molecular techniques, techniques focused on metabolism, and staining techniques. The molecular techniques are based on detecting individual and global gene expression in non-culturable cells. According to the literature, the use of transcripts is an excellent alternative, given that mRNA half-lives are typically in the range of seconds to minutes [104,105] and the identification of the mRNA is evidence that cells remain metabolically active. In addition, their detection provides an essential insight into the factors that may be regulated in the VBNC sate. The techniques focused on metabolism search for metabolites or enzymatic activity, generally using methodologies with colorimetric results, biosensors, or matrices. The detection of metabolic activity in bacteria in a viable but non-culturable state serves as an indicator of cell viability. This detection signifies that despite the bacteria’s inability to be cultured under standard laboratory conditions, there is biological activity. Evidence indicates that bacteria in the VBNC state retain the ability to conduct fundamental metabolic processes, including respiration, nutrient assimilation, and gene expression (see Section 5 and Section 6). The staining techniques mainly are based on detecting activity in the electron transport system and the integrity of the cytoplasmatic membrane. Cellular respiration allows the rapid identification of metabolically active cells, only using a compound fluorescent or a compound that reacts and forms a fluorescent compound. For example, CTC is reduced to a red fluorescent compound called formazan. The BacLight^®^ Live/Dead Kit is a tool that is frequently used, in which, by differential staining and fluorescence microscopy, bacteria in the VBNC state stain green, indicating that they have largely intact membranes and thus can be considered to be alive [16]. However, in the case of bacteria under desiccation stress, the membranes suffer apparent sublethal damage during the VBNC state and they stain red [4]. On the other hand, it has been reported that some dead bacteria are observed as empty particles lacking cytosol that do not allow propidium iodide entry, but their membranes remain intact. This type of situation can lead to the erroneous interpretation of the results [106]. It is important to note that there is not a decisive test for bacterial viability in the VBNC state, and it is usually recommended that, when trying to prove this fact, two or more methodologies should be performed. Arvaniti and colleagues [107] have raised concerns that the employed method could potentially result in a misinterpretation of the VBNC state. The integration of various approaches enables the assessment of multiple parameters, such as metabolic activity and membrane integrity, ensuring more reliable determination. Interestingly, although several techniques have been developed to elucidate the VBNC state, their application in beneficial bacteria is limited. The most commonly used techniques include Live/Dead Baclight staining, CTC, DFA-DVC, RT-qPCR, and qPCR (Figure 3).

## 5. What Happens during the VBNC State?

There are different characteristics shown by cells in the VBNC state. Several bacterial species decrease their size, producing several metabolic changes, including the depletion of energy reserves, altered gene expression, and DNA replication [3]. Biosynthesis is a process that does not stop during this state, with cells forming new proteins related to starvation and cold shock [108,109]. ATP levels rapidly decrease in dead cells; however, in VBNC cells, these levels are high [110]. Pazos-Rojas et al. (2019) detected the expression of the *opr*H, *mut*S, and *mut*L genes and 16S RNA in *P. putida* KT2440 VBNC cells under desiccation stress [4]. These studies suggest that some genes may be effective monitors for viability, but whether they are involved in the entrance or exit from the VBNC state is still unknown.

At the structural level, several characteristics have been observed in non-culturable cells [111], including thickened cell envelopes that potentially provide enhanced resistance to various stressors, contributing to their sustained viability. Changes in the cell membrane composition aid in maintaining ionic homeostasis and, consequently, the intracellular water content. A granular cytoplasmic appearance may indicate reduced ribosome numbers or protein aggregation. Nucleoid compaction likely plays a role in preserving the genome more effectively. Recent proposals suggest that alterations such as cellular structure reorganization, global protein aggregation, and ribosome dimerization may facilitate the transition of bacteria into the VBNC state when exposed to different types of stress [112].

At the DNA level, three types of stable condensation can form: nanocrystalline structures, liquid crystalline structures, and a structure similar to a folded nucleosome. This type of rearrangement may be the result of complex interactions and associations of proteins to protect the integrity of the genome [113]. Loiko et al. (2017) [114] reported that the crystallization of nucleoids in complexes with DNA-binding protein of starvation (Dps) or small acid-soluble proteins (SASP) can offer protection against damaging factors, representing a necessary form of structural organization for DNA in VBNC microorganisms. These discoveries could elucidate the long-term survival mechanisms in conditions inhibiting growth, thereby facilitating species preservation.

In the cytoplasmic membrane, it has been described that VBNC cells may suffer damage and modifications in the composition of fatty acids, strongly suggesting that changes may be essential for entrance into this state [4,115]. Cells may undergo biochemical changes in cell walls, and genes involved in peptidoglycan biosynthesis could be a necessary characteristic of VBNC cells to create a more rigid wall than in actively dividing cells [116].

Bacteria can detect stressors through histidine kinases bound to the membrane, mediating the cellular response through the differential expression of the target genes [117]. Therefore, early signaling could be a determining factor to induce the VBNC state in bacteria. As mentioned above, cellular energy is one of the key characteristics of the VBNC state, triggering the induction of genes codifying subunits of proton pumps such as NADH, or ubiquinone oxidoreductase, a protein that is essential for processes requiring energy in VBNC cells as well as under normal conditions [118]. This suggests that when bacterial cells enter the VBNC state in response to an environmental change, the activity of complex I of the respiratory chain and the NADPH-generating systems are critical for the maintenance of cell viability [118]. In addition, selective permeability to nutrients and metabolites provided by ABC transporters may be a prerequisite for the VBNC state [119]. Although this understanding has been developed within pathogenic bacteria, it is possible to consider that this behavior is widely distributed among beneficial bacteria.

## 6. Proteomics, Transcriptomics, and Metabolomics of Bacterial Cells in VBNC State

When bacteria enter the VBNC state, various cellular changes may occur. Studying these changes at the post-transcriptional and post-translational levels could elucidate which genes are important for bacteria to perform this phenomenon; however, knowledge is still limited under the VBNC state. It has been observed that under various types of stress inducing the VBNC state, different genes may be expressed—for instance, more alkaline phosphatase and α-ketoglutarate oxidoreductase [120]; an increase in OmpW external membrane proteins [121]; the high expression of glutathione S-transferase [122]; the overexpression of several proteins related to transcription, translation, ATP synthesis, and gluconeogenesis; and antioxidants [123].

The expression of the RpoS sigma factor in the VBNC state has been demonstrated in several bacterial species, with guanosine 3′, 5′-bispirophosphate (ppGpp) acting as a positive regulator during the synthesis and function of this sigma factor [124]. Interestingly, it has been observed that RpoS mutant strains quickly lose cultivability and cannot return to the culturable state with existing resuscitation methods [124,125]. Considering that the absence of this protein can mean imminent cell death, it could be one of the key genes involved in the entrance and exit from the VBNC state in bacteria.

The activity in the ribosomes is one of the most important processes in all living cells. A low expression level for genes encoding ribosome-associated inhibitor A (RaiA) proteins has been observed in the VBNC state for several bacterial species [125]. This could mean that bacteria decrease their translational activity during the latency status, thereby saving energy in order to face stress conditions.

It is likely that the entrance into this state begins with metabolic pathways that control stimulus response mechanisms (two-component systems) and bacterial movement (chemotaxis), determined by the presence of chemicals in the environment where the cells are located [119]. Furthermore, the VBNC state in some bacteria could also be regulated by the proteins leucine-responsive regulatory protein/asparagine synthase C products (Lrp/AsnC) and MarR [119]. The MarR protein controls genes involved in the degradation of toxic compounds (including phenols), virulence, the export of harmful chemicals, and resistance to oxidative stress [126].

Within the scant literature on beneficial bacteria, there are studies on *P. putida* KT2440 and *C. metallidurans*. Transcriptomic studies of *P. putida* KT2440 in the VBNC state caused by desiccation stress [127] showed that six genes related to transmembrane transport and oxidation-reduction processes were upregulated. The ethylene glycol porin (PP_2662) and substrate-binding protein (PP_2676) genes could transport and degrade polyhydric alcohols, which are accumulated during desiccation stress as compatible solutes. The upregulation of the TonB-dependent receptor (PP_1446) gene could be a strategy used by *P. putida* KT2440 to provide the ferrous iron required for vital functions during the VBNC state. *P. putida* KT2440 cells return to a cultivable state upon 24 h of rehydration; after this return, 148 genes related to transport, oxidation-reduction, the regulation of transcription, and biosynthetic processes were upregulated, while 42 genes related to translation, oxidation-reduction, and the regulation of transcription were downregulated. During the prolonged rehydration of *P. putida* KT2440 cells, the catabolism of phenylalanine/tyrosine is activated, possibly to provide energy and a carbon source for ubiquinone biosynthesis, while maintaining reduced protein synthesis [127].

Studies in *C. metallidurans* show that during the transition from the culturable state to the VBNC state, there is a strong decrease in the expression of proteins involved in different mechanisms, such as basal bacterial metabolism, cellular processes, signaling, information storage, protein synthesis pathways, energetic processes, and cell shape regulation [49]. The proteins that show an increase in their regulation are involved in energetic processes and redox reactions. The proteomic analysis of the return to a culturable state showed that gluconate increased the expression of several proteins related to fundamental bacterial metabolism when used. Meanwhile, when only water was added to promote the return to the culturable state, the expression of only six proteins was increased. These results suggest that reduced soil carbon or water availability could initiate the bacterial VBNC state in soil-like environments. This limitation could induce gene expression and protein synthesis, leading to the VBNC state.

In recent metabolomics studies of the VBNC state, it has been observed in various bacterial species that active metabolic pathways are linked to the transport and metabolism of inorganic ions, carbohydrates, and amino acids, as well as pathways involved in the synthesis of the cell wall, membrane, and envelope [128]. Additionally, it has been determined that during the VBNC state, there is an augmentation in the amino acid content, consequently leading to the alteration of specific amino acid metabolic pathways, such as the biosynthesis of aminoacyl-tRNA and the metabolism of arginine, proline, alanine, aspartate, and glutamate. Additionally, shifts were observed in D-glutamine and D-glutamate metabolism, β-alanine and arginine biosynthesis, and the valine, leucine, and isoleucine degradation pathways Another notable metabolic change was a substantial increase in cAMP content, which could potentially function as an inducing factor for the VBNC state [129]. Other studies suggest that the glyoxylate cycle serves as a pivotal metabolic pathway for stress resistance and in maintaining cellular metabolic balance during the VBNC state [130].

The metabolomic analysis of *Lacticaseibacillus paracasei* in the VBNC state revealed 25 differential metabolites from five major classes: amino acids, carbohydrates, lipids, vitamins, and purines and pyrimidines. The levels of L-cysteine, L-alanine, L-lysine, and L-arginine increased markedly in the cells that returned to the culturable state, while the levels of cellulose, alginose, and guanine decreased significantly. Cells that left the non-culturable state had higher levels of cysteine, L-glutamic acid, L-arginine, and L-glutamine. A compound identified at high levels was xylooligosaccharide, which probably favors the return to the cultivable state [129].

It is important to note that a universally applicable molecular or metabolic mechanism explaining the VBNC state has not yet been fully developed. Based on the above evidence, the only assertion that we can make is that bacteria exhibit a broad spectrum of possibilities and strategies to enter, persist in, and exit the culturable state. These behaviors will depend on various factors: the specific stress triggering this state, the mechanisms facilitating its reversal, and the characteristics unique to each bacterial species.

## 7. Resuscitation of Bacteria under VBNC State

As mentioned in the previous sections, bacteria in the viable but non-culturable state do not grow in routine bacteriological media. However, they are still alive; thus, this state may constitute a survival strategy under stressful conditions. The cells in the non-culturable state must be able to increase their metabolic activity to return to the culturable state [2]. Numerous studies have demonstrated that the return to a cultivable state becomes possible upon removal of the stressor. It has been a topic of discussion and a great challenge to demonstrate that cells can return to the culturable state and that observed cells that grow again on media do not simply correspond to the growth of other surviving bacteria.

In *P. putida* KT2440 cells in the VBNC state induced by desiccation, the colonization of the rhizosphere of maize plants, short rehydration in the presence of root exudates, and prolonged rehydration with only distilled sterile water were shown to allow the return to a culturable state [4].

Interestingly, it has been observed that interaction with higher organisms can function as a biological mediator in the return to the culturable state. For example, in *L. pneumophila*, which enters the VBNC state under starvation and hypochlorite treatment, it can return to the culturable state in the presence of protozoa *Acanthamoeba polyphaga* and *Acanthamoeba castellanii* [131,132]. Regarding beneficial bacteria, a recent study has shown that VBNC cells of *P. putida* KT2440 can colonize the rhizosphere of maize plants and this interaction allows the return to a culturable state [4]. Furthermore, the root exudates of maize can return these bacteria to the culturable state [4]; thus, it is of interest to study bacterial associations with their hosts, since this influences the survival of the bacteria even in the VBNC state.

Other factors that could be decisive for the return to the culturable state are based on the study of extracellular proteins such as the resuscitation promoting factor (Rpf) [133] and the YeaZ protein with protease activity [134,135]. The promoting effect of YeaZ may be correlated with its protease activity, but the mechanisms that help in the recovery of cultivability need further investigation.

Additionally, certain mechanisms have been studied to facilitate the return to the cultivable state. These include the activity of a peptidoglycan hydrolase involved in the digestion of the cell wall and cell division [116,134,136] and the “autoinducer of growth”, which were found to be stable to heat and were secreted by Gram-positive and Gram-negative bacteria growing in culture media with the hormone norepinephrine [137]. It would be interesting to determine whether beneficial bacteria in the VBNC state are also susceptible to resuscitation by these autoinducers.

Molecules such as sodium pyruvate have been studied for their role in the return to a culturable state. This molecule is an intermediate key metabolite in glycolysis and a H_2_O_2_ degrading compound [138]. It was observed that VBNC cells can return to a culturable state in media supplemented with sodium pyruvate [139]. It was suggested that sodium pyruvate, catalase, and superoxide dismutase, due to their H_2_O_2_ or reactive oxygen degrading effects, can induce the return to a cultivable state [140]. It has been proposed that cells in the VBNC state could utilize their remaining ATP to synthesize NAD+, primarily aimed at restoring the energy production machinery and reviving cellular metabolic activity. This mechanism facilitates activity at a transduction level, promoting resuscitation processes, essentially directing their resources towards restarting energy production as a precursor to subsequent resuscitation [141]. The molecular mechanisms governing the initiation of resuscitation, including the detection of signal molecules associated with resuscitation and the transduction of these signals to subsequent events, remain unclear and should be explored in future research on the VBNC state.

Quorum sensing (QS) is a widespread communication system in bacteria that induces global gene expression changes [142]. It has been observed that QS can induce an adaptation to stressful conditions and plays a role in the return from the VBNC state [143,144]. It is proposed that QS may help the cell to express genes related to oxidative stress, which, as mentioned above, play an important role in allowing return to the culturable state in different bacteria, including beneficial bacteria. It is certain that the VBNC state is reversible, which opens up a range of possibilities for the use of microorganisms in different areas that may offer some benefit.

## 8. Conclusions

The study of the VBNC state in bacteria remains controversial, because its verification does not completely satisfy all researchers worldwide, although many scientists have concluded that this is a survival strategy under stressful conditions. Regardless of the role of the VBNC state in the life cycle of bacteria, it has been fully proven that many bacterial species, especially human pathogens, can carry out this survival strategy. During the VBNC state, bacteria maintain their cellular structures and biological functions, such as cellular respiration and continuous gene expression, having the ability to leave this state and return to a culturable state when the conditions become more favorable. Despite the advances made over the last thirty years, it remains a challenge to investigate this state’s physiology, biochemistry, and genetics, because it is unknown which gene or genes are directly involved in the loss and recovery of cultivability. The study of beneficial bacteria in the VBNC state is still lacking. Expanding knowledge in this field could represent an opportunity for future biotechnological applications—for example, for the biological control of plant pathogens, since, even when bacteria enter the VBNC state under stressful conditions, when returning to the culturable state, they can continue exercising their biocontrol over species harmful to their hosts. Microbial biocontrol technology avoids the use of pesticides that are highly toxic to the environment. Another application is the formulation of bacterial inoculants with beneficial species capable of entering the VBNC state, to increase their shelf life, so that, when applied to the plant rhizosphere, the bacteria will be able to return to the culturable state and carry out their beneficial activity on the growth of plants of agricultural interest. The impact of beneficial bacteria on human health represents a challenge in terms of understanding the role of the VBNC state and how it can affect or benefit hosts. Meanwhile, the elaboration of consumable biotechnological products using only culturable microorganisms is a major challenge for the industry; however, we must understand that bacteria with a preservation system, such as the VBNC state, can offer a significant decrease in production costs. This highlights the importance of more precisely understanding the transition between the culturable and non-culturable state.

## Figures and Tables

**Table 1:** Main species of beneficial bacteria that enter the viable but non-culturable state.

Group of Bacteria		Species	Conditions that Induce VBNC State	References
*Proteobecteria*	*Alphaproteobacteria*	*Acetobacter aceti*	Treatment with SO_2_ at a concentration of 30 and 50 mg/L	[46]
		*Acetobacter pasteurianus*	High acid stress during fermentation	[47]
		*Methylosinus sporium*	Freeze drying and cryopreservation (liquid nitrogen)	[25,30]
		*Methylosinus trichosporium*		
		*Methylocystis hirsuta*		
		*Methylocystis parvus*		
		*Methylocella tundrae*		
		*Rhizobium leguminosarum*	Cupric sulfate to a concentration of 60 ppm	[48]
		*Sinorhizobium meliloti*	Incubation at 25 °C in tap water (microcosm-water) Incubation under anoxic conditions in liquid microcosms Incubation in nitrocellulose filters at relative humidity of 22% for three days at 20 °C in the dark	[16]
	*Betaproteobacteria*	*Cupriavidus metallidurans*	Incubation in artificial soil at 30 °C for 12 days, without any C source or H_2_O	[49]
	*Gamaproteobacteria*	*Methylomonas methanica*	Lyophilization and cryopreservation (liquid nitrogen)	[25,30]
		*Methylosarcina fibrata*		
		*Methylocaldum gracile*		
		*Methylomicrobium alcaliphilum*		
		*Methylococcus capsulatus*		
		*Microbulbifer aggregans*	Incubation in modified artificial seawater (ASW) for 4 h at 30 °C	[50]
		*Pseudomonas fluorescens*	Incubation in saline solution (NaCl 0.9% *w*/*v*) at 37 °C Exposure to benzalkonium chloride (BAC)	[51]
		*Pseudomonas putida* KT2440	Desiccation at 30 °C and 50% relative humidity	[4]
		*Vibrio fischeri*	Incubation at 22 °C in nutrient-limited artificial seawater (ASW)	[52]
*Terrabacteria*	*Actinobacteria*	*Bifidobacterium animalis* subsp. *lactis*	Storage in fermented foods Refrigerated storage of butter for 4 weeks Microcapsules with full-fat goat milk and inulin-type fructans	[53]
		*Bifidobacterium longum*	Storage in fermented foods	[54]
		*Arthrobacter albidus*, reclassified as *Sinomonas albida*	Absence of resuscitation promoting factor (Rpf) protein in the culture medium	[55,56]
	*Firmicutes*	*Bacillus coagulans*	Incubation at pH 2 for 24 h and subsequent incubation at 140 °C for 5 min	[57]
		*Lactiplantibacillus plantarum*	Treatment for 30 min at 100 °C or with 1 mol/L HCl Incubation in beer at 0 °C temperature	[20]
		*Oenococcus oeni*	Sulfur dioxide and histidine decarboxylase activity in wines	[46]
